# A Single Voice Prosthesis Utilized For 17 Years

**DOI:** 10.22038/ijorl.2019.30795.2007

**Published:** 2019-11

**Authors:** Ahmet Mutlu, Erdem Koroglu, Selvet Erdogan

**Affiliations:** 1 Department of Otorhinolaryngology, Istanbul Medeniyet University Goztepe Research and Training Hospital, Istanbul, Turkey.; 2 Department of Otorhinolaryngology, Kocaeli University Medical Faculty, Kocaeli, Turkey.

**Keywords:** Biofilms, Tracheoesophageal puncture, Voice prosthesis

## Abstract

**Introduction:**

Surgical procedures, especially total laryngectomy, have a profound adverse effect on the patient's physical, functional, as well as emotional health, and strongly decrease quality of life. Tracheoesophageal puncture is one of the most significant method that is widely performed successfully by physicians. Current valve technology enables long expiring duration; however, general duration for all types of valve appears to be approximately 3 to 6 months.

**Case Report:**

A 72-year-old patient with total laryngectomy and tracheoesophageal voice prosthesis (VP) presented our voice clinic with difficulty in swallowing and leakage around the valve of VP. In this report, we aim to present the patient who has used a single voice prosthesis for 17 years without a complication.

**Conclusion:**

In our case, the nutritional habits of our patient may have allowed him to use VP for 17 years without complications. We strongly advise following the suggested renewal time of voice prosthesis.

## Introduction

Surgical procedures, especially total laryngectomy, have a profound adverse effect on the patient's physical, functional, and emotional health, and strongly decrease quality of life. Voice reconstruction modalities are a tracheoesophageal puncture, esophageal speech, and electrolarynx. Tracheoesophageal puncture is one of the most significant methods that is widely performed successfully by physicians. The voice prosthesis (VP) device life is a limiting factor of tracheoesophageal speech. General knowledge of the prosthesis life appears to be approximately 3 to 6 months. In fact, current valve technology enables long expiring duration with anti-microbial structures (1). On the other hand, it is currently debated that the device life for all types of tracheoesophageal voice prosthesis may not be as long as represented in literature (2). In this report, we aim to present a patient who has surprisingly used a single voice prosthesis for 17 years without a complication. 

## Case Report

A total laryngectomized 72-year-old male was admitted with a complaint of difficulty in swallowing the foods and intractable liquid leakage from the valve of the tracheoesophageal prosthesis. The examination revealed a prosthesis covered with crust and dry mucus. Patient history was obtained from medical records and it was revealed that he had been using the same voice prosthesis smoothly for 17 years without changing. In fact, he had no significant complication in this period. However, he had been experiencing some difficulties in swallowing and leakage around the valve for a year. In addition, he had a serious iatrophobia and refused the change of prosthesis for several times.

**Fig 1:**
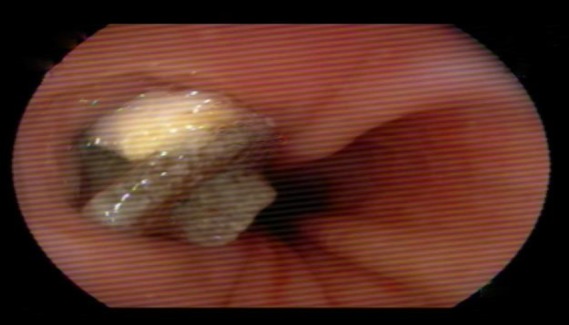
The tracheoesophageal voice prosthesis in the upper esophagus during trans oral fiberoptic endoscopy. No lesion was detected on the esophageal mucosa

**Fig 2:**
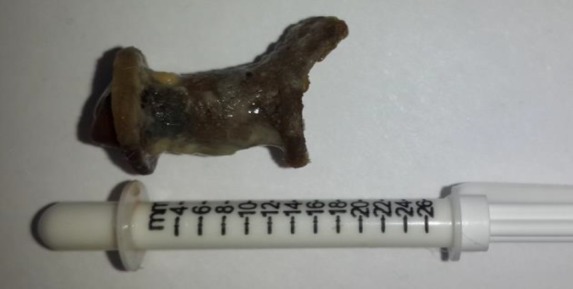
The old voice prosthesis covered with crust and dry mucus

He was questioned for his daily habits which might help to preserve the prosthesis for a long time Tracheoesophageal fistula construction was performed after total laryngectomy for voice and speech restoration. Furthermore, flexible fiber optic esophagoscopy was performed to evaluate the fistula in case of a lesion; however, no lesion was detected on the esophageal mucosa (Fig.1) (3). The patient was referred to a psychologist to make him convinced regarding the prosthesis change. After the removal of the old prosthesis (Fig.2), a new voice prosthesis (20-F,16 mm) was inserted and no complication occurred.

## Discussion

Voice prosthesis application needs the creation of tracheoesophageal fistula in laryngectomized patients. The fistula may be performed at the time of laryngectomy or a secondary procedure. As it was known, the prosthetic speech was an indispensable approach for laryngectomized patients with simple surgical methods, short training period, and immediate phonation. 

On the other hand, some complications were reported ranging from mild to severe (i.e., leakage, paraesophageal abscess, mediastinitis) and intractable leakage was the most prevalent complication (4). Leakage was one of the main complications which determine the device life (2). In fact, microbial contamination and colonization (i.e., bacterial, fungal) might cause the intractable leakage through the prosthesis valve with the biofilm formation (5). It was still a debate of reducing the microbial burden on the VP except the mechanical cleaning. Dairy products (e.g., buttermilk) consumption was one of the reported options. The acidic nature (pH: 4.5) of the buttermilk and viable probiotic content could be the reason for antimicrobial effect (5). Vinegar might be another effective acidic agent for the prolonged lifetime of the prosthetic devices (6). Proper antimicrobial drug regimen might also enhance the expiring time; however, the development of more resistive strains was a great handicap of this option (1,5). We present a patient who has lived with the same prosthetic device for 17 years. When the patient was queried about his daily life and habits, he mentioned daily consumption of yogurt, and ayran, which is a drink made from yogurt and is the same as buttermilk. Furthermore, the patient had habitually consumed fresh salad with vinegar or lemon, which are mildly acidic foods. Moreover, he had frequently cleaned the crusts on the tracheal portion. Those were not certain evidence to explain this situation and might be considered unrealistic; however, the patient managed to live with a single VP for 17 years. 

## Conclusion

In our case, the nutritional habits of the patient may have allowed him to use VP for 17 years without complications. We strongly advise following the suggested renewal time of voice prosthesis.
